# Chinese patent medicine for osteoporosis: a systematic review and meta-analysis

**DOI:** 10.1080/21655979.2022.2038941

**Published:** 2022-02-19

**Authors:** Yan Jia, Jigao Sun, Yan Zhao, Kaiqiang Tang, Ruizheng Zhu, Wei Zhao, Rongtian Wang, Yanqiong Zhang, Na Lin, Weiheng Chen

**Affiliations:** a Department of Minimally Invasive Arthrology, The Third Affiliated Hospital of Beijing University of Chinese Medicine, Beijing, China; b Department of Orthopedics, Dongfang Hospital Beijing University of Chinese Medicine, Beijing, China; c Department of Orthopedics, Luoyang Hospital of Traditional Chinese Medicine, Luoyang, China; d Institute of Chinese Materia Medica, China Academy of Chinese Medical Sciences, Beijing, China

**Keywords:** Chinese patent medicine, clinical efficacy, herbal medicine, meta-analysis, osteoporosis

## Abstract

Chinese patent medicine (CPM) has been widely used in China for patients with osteoporosis (OP) but a comprehensive literature review is still important. Therefore, we performed meta-analysis using six electronic databases prior to 30 April 2021 only randomized controlled trials (RCTs) using CPM as the first-line treatment in adults with OP were included. Thirty RCTs met the inclusion criteria with a total of 2723 patients, and seven types of CPM were included. Compared with the control group, 23 studies showed significantly improved bone mineral density (BMD) (lumbar spine) (mean difference [MD] = 0.08; confidence interval [CI], 0.03 to 0.13), 15 studies showed significantly improved BMD (femoral) (MD = 0.05; 95% CI, 0.02 to 0.07), 6 studies showed significantly improved BMD (radius) (MD = 0.06; 95% CI, 0.03 to 0.09), 2 trials showed significantly improvement of BMD (ulna) (MD = 0.02; 95% CI, 0.01 to 0.03), and 4 trials showed significantly improved BMD (MD = 0.09; 95% CI, 0.09 to 0.10). The meta-analysis also showed that CPM had superior pain improvement, a higher total effectiveness rate, and a lower risk of adverse events compared with standard western treatment. The findings of this study suggest that CPM therapy may be a safe and effective alternative treatment modality for OP, it has potential benefits in relieving symptoms and improving BMD compared to western medications or placebos.

## Graphical Abstract

**Figure:**
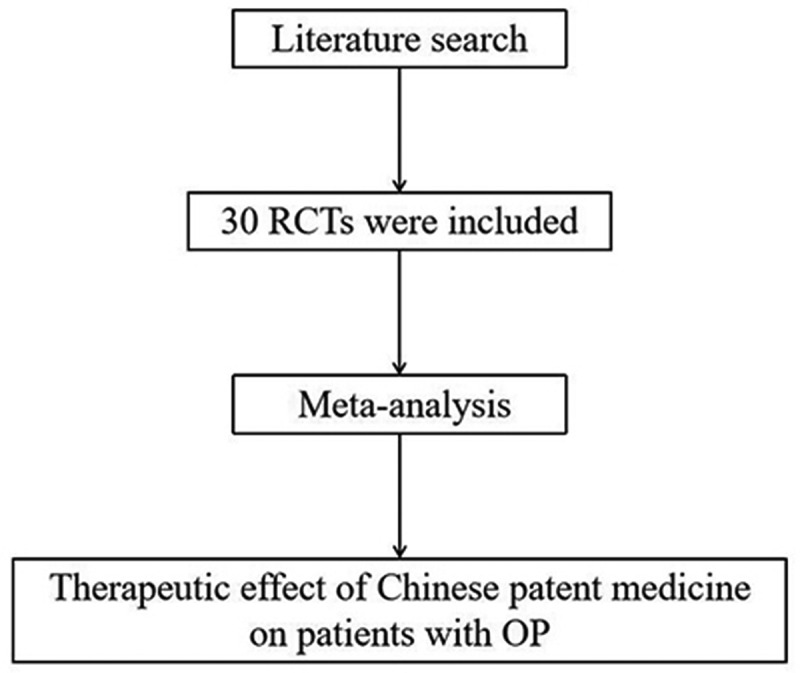

## Introduction

Osteoporosis (OP) is a systemic skeletal disease characterized by decreased bone mass and microarchitectural deterioration of bone tissue, resulting in increased bone fragility and fracture risk [1]. OP imposes exorbitant financial expenditures on society, while patients suffer from serious bone fractures and physical agony [2]. According to reports, the prevalence of OP has grown, and it now affects 34.65% of adults aged over 50 years in China [3]. There are also several therapies for OP, such as bisphosphonates, which are the most often recommended drug for the illness [4]. Bisphosphonates, on the other hand, are linked to a number of possible dangers, including osteonecrosis and gastrointestinal side effects [5]. As a necessary consequence, there has been an upsurge in discovering methods to prevent and cure OP.

Traditional Chinese medicine (TCM) is heavily favored in the treatment of OP in China. According to TCM theory, OP is classified as ‘bone impediment’ or ‘bone wilting’ caused by an insufficient innate endowment and an imbalance of acquired absorption and nourishment. TCM theory asserts that an invasion of exogenous evil can induce OP, leading to a disharmony of yin-yang, qi, and blood; a deficiency of the spleen, liver, and kidney; and a loss of bone nourishment [6]. Correspondingly, the principle of Chinese medicine treatment is to tonify the kidney and strong bones. Chinese patent medicine (CPM) is composed of Chinese herbal medicines as raw materials and processed into TCM products according to the prescribed prescription and preparation process [7]. CPM includes various forms such as pills, powders, granules, and capsules [8]. Currently, there are hundreds of types of CPM used for the treatment of OP, and several recent studies have suggested that their active ingredients may exert a certain effect on bone mineral density (BMD) and overall symptoms by increasing hormone levels and regulating bone metabolism-related pathways [9,10]. Although CPM has long been regarded as a key component in China and recommended in several Chinese treatment guidelines of OP, either as a monotherapy or in combination with standard western medicine, the quality of the evidence has led to varying degrees of efficacy and safety assessments. Many new clinical studies have been published since then, but existing systematic reviews were still limited by samples, methodological quality [11,12], or specific kinds of CPM [13].

From this, it can be seen that a comprehensive review is still an important step for making recommendations in clinical practice. Thus, we systematically reviewed the a large amount of medical literature and performed a meta-analysis on randomized controlled trials (RCTs) of CPM therapy for patients with OP to understand its benefits for OP.

## Methods

### Protocol and registration

This study was based on the recommendations of the Cochrane handbook for systematic reviews of interventions and reported according to the Preferred Reporting Items for Systematic Reviews and Meta-Analyses (PRISMA) statement [14]. This study has been registered on PROSPERO (CRD42020183795).

### Search strategy

We searched the following six electronic databases to identify qualified trials published from inception to 30 April 2021: PubMed, Cochrane Library, Chinese Biomedical Databases (CBM), Chinese National Knowledge Infrastructure (CNKI), Wan Fang, and Chongqing VIP. In addition, we manually searched for publication records from the library. There were no restriction on publication language. The search strategy included the following keywords: traditional Chinese medication, traditional Chinese patent medicine, capsule, tablet, powders, pill, granules, osteoporosis, clinical trial, and randomized controlled trial.

### Eligibility criteria

We only included RCTs that compared CPM with conventional western therapies and placebos for the treatment of OP and that involved interventions of CPM therapy for the duration of at least 2 weeks with more than 10 subjects in each group. The diagnostic criterion was from the OP Committee of Chinese Gerontology Society [15,16] and Chinese Medical Association [17,18]. We also accepted diagnostic criteria for primary OP in Chinese (Trial) [19]. To be eligible for this study, the experimental group had to be treated with CPM, and the control group had to only receive non-Chinese medicine interventions, such as calcium, alpha calcidol, or alendronate. There was no language restriction in document retrieval. We excluded review articles, theoretical research, case reports, animal experiments, and any control group that included traditional Chinese therapies.

### Study selection

Two authors independently screened all potentially eligible studies. Titles and abstracts were first screened to exclude irrelevant citations. The full text of all potential articles was retrieved and screened according to the study qualification criteria. Disagreements were resolved by consensus or discussion with a third author.

BMD was the first outcome in this study to evaluate the clinical efficacy of CPM in the treatment of OP, including lumbar BMD, femoral BMD, ulna BMD, and radius BMD. We also used pain level and total effectiveness rate to measure the effects of CPM on clinical symptoms. Pain level was measured using the visual analogue scale (VAS), and the VAS score ranged from 0 point (no pain) to 10 points (worst possible pain), where a lower score indicates a better outcome. The total effectiveness rate [20] was used to evaluate overall pain, physical performance, and wellness. The total effectiveness rate was assessed based on the number of patients in each of the following categories: ‘Clinically cured,’ (the pain and swelling of joints had disappeared and active function had returned to normal); ‘Significant improvement,’ (the pain and swelling of joints was alleviated and active function had improved significantly); ‘Improvement,’ (the pain and swelling of joints was partially alleviated and active function had improved); and ‘Not cured,’ (the pain and swelling of joints remained unchanged and there was no improvement of active function).

### Data collection and quality assessment

A pre-designed data extraction table was used to extract data from the selected studies, including publication information, gender, age, interventions, control measures, outcomes, summary of results, and adverse reactions. One author evaluated all data extraction and quality ratings for consistency and resolved discordant responses.

### Statistical analysis

All analyses were conducted using RevMan V5.3 (The Nordic Cochrane Center, The Cochrane Collaboration), and study quality was assessed using the Cochrane Risk of Bias Tool. For meta-analysis of BMD and pain score, we combined studies using mean difference (MD) or standard mean difference (SMD) in the BMD score and VAS score. We calculated 95% confidence interval (CI) based on the mean change from baseline to the study endpoint, and we evaluated heterogeneity using the *I*^2^ statistic. The fixed-effect model was used if *I*^2^ < 50%, otherwise a random-effects model was applied. For a meta-analysis of the total effectiveness rate, we combined studies using risk ratio (RR) comparing CPM therapy with controls. P-value < 0.05 was considered to be statistically significant for all results.

## Results

### Brief introduction

A comprehensive review is still an important step in developing clinical practice recommendations. Thus, we systematically reviewed the prior medical literature and performed a meta-analysis on randomized controlled trials (RCTs) of CPM therapy for patients with OP to better understand its benefits for OP. We searched electronic databases for qualifying publications before extracting pertinent data for meta-analysis. Finally, the results showed that the CPM improves therapeutic impact while having less side effects when compared to typical western therapy.

### Study selection

**Figure 1.**
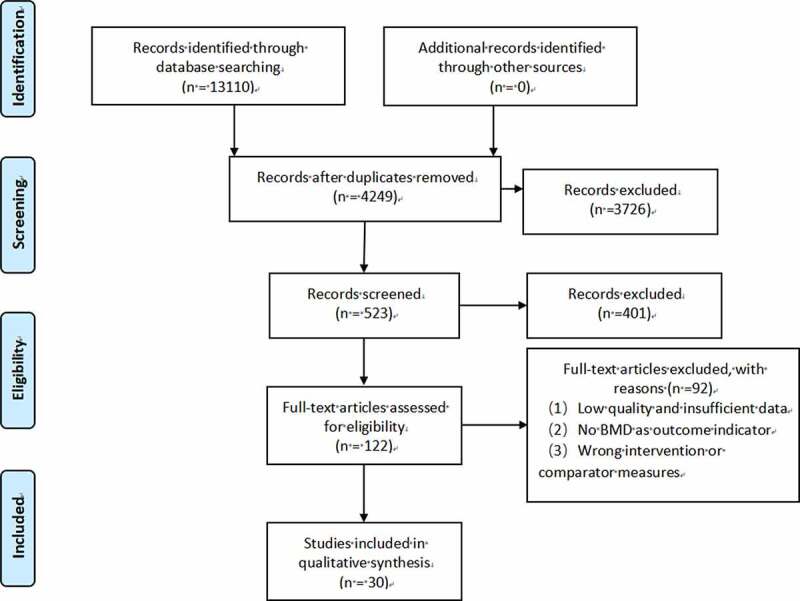
Flow diagram of literature search.

We screened a total of 13,110 studies from 6 databases. Following an initial review of 523 possibly relevant abstracts, we excluded 401 abstracts because they did not match the inclusion criteria. We retrieved and reviewed 122 full articles, and 92 articles were excluded due to low quality, insufficient data, no outcome of BMD, wrong intervention, or comparator measures. Finally, thirty studies [21–50] published between 2004 and 2020 were included. Only one study was published in English. Figure 1 summarizes the detailed study selection process.

### Study characteristics

The characteristics of the 30 trials are summarized in Table 1. All 30 RCTs with a total of 2723 people were carried out in China, and the total sample size of included RCTs ranged from 39 to 200 (median: 81). The participants varied in age from 47 to 75 years (median: 61.5 years), with women accounting for 32.93% to 100% (average: 74.95%) of the total. Table 2 summarizes the evidence and major impact of CPM therapies for OP.

**Table 1.** Characteristics of 30 included studies of CPM for osteoporosis

Author, (yr)	N (Female, %)	Age (yr)	CPM	Controls	Outcomes Assessments	Results (CPM vs. Control)	P value	Adverse Events (CPM vs. Control)
**1. Jintiange capsule**								
Yuan, 2019	160 (NR)	68	Jintiange capsule, 3 capsules/time, 3 times/day, 12wks	Caltrate D, 600 mg, once/day, 12wks	BMD (LS)	0.728 vs 0.535	< 0.05	NR
Zeng M, 2016	86 (100%)	59	Jintiange capsule, 2 capsules/time, 2 times/day, 24wks	Alendronate sodium, 70 mg, once /week, 24wks	BMD (radius)	0.268 vs 0.269	> 0.05	NR
Qin, 2016	112 (100%)	63	Jintiange capsule, 3 capsules/time, 3 times/day, 12wks	Caltrate D, 1 capsule/time, once/day, 12wks	BMD Total effectiveness rate	0.62 vs 0.51 95.6 vs 73.2	< 0.05 < 0.05	NO
He, 2015	160 (57.5%)	65	Jintiange capsule, 3 capsules/time, 3 times/day, 36wks	Caltrate D, 600 mg, 2 times/d, 12wks	BMD (LS) BMD (femoral)	0.618 vs 0.5355 0.5855 vs 0.5315	< 0.01 < 0.01	8 vs 8
Cai, 2015	64 (100%)	61	Jintiange capsule, 1.2 g/time, 3 times/day, 24wks	Alendronate sodium, 70 mg, once/week, 24wks	BMD (LS) BMD (femoral)	0.9 vs 0.9 0.9 vs 0.9	> 0.05 > 0.05	0 vs 3
**2. Qianggu capsule**								
Zhao, 2004	69 (100%)	56	Qianggu capsule, 1 capsule/time, 3 times/day, 24wks	Livial, 1.25 mg, once/day, 24wks	BMD (LS)	0.781 vs 0.826	> 0.05	3 vs 8
Wang, 2007	54 (100%)	62	Qianggu capsule, 1 capsule/time, 3 times/day, 24wks	α-D3 capsule, 1 capsule/time, twice/day, 24wks	BMD (LS) BMD (femoral) Total effectiveness rate	0.797 vs 0.762 0.693 vs 0.656 92.9 vs 88.5	< 0.01 < 0.01 < 0.05	3 vs 0
Ji, 2006	62 (70.97%)	65	Qianggu capsule, 1 capsule/time, 3 times/day, 12wks	Vitamin D2 calcium hydrogen phosphate tablets, 2 tablets/time, 3 times/day, 12wks	BMD (ulna) BMD (radius) Total effectiveness rate	0.62 vs 0.565 0.625 vs 0.545 97 vs 90	< 0.05 < 0.05 < 0.05	NR
Gu, 2004	82 (32.98%)	63	Qianggu capsule, 1 capsule/time, 3 times/day, 12wks	Calcium gluconate, 3 tablets /time, 3 times/day, 12wks	BMD	0.742 vs 0.684	< 0.05	NR
Wang, 2019	132 (100%)	58	Dizhong Qianggu capsule, 3 capsules/time, 3 times/day, 48wks	Alendronate sodium, 70 mg, once/week, 24wks	BMD (LS) BMD (femoral) Total effectiveness rate VAS pain	0.836 vs 0.842 0.753 vs 0.757 84.85 vs 77.27 1.9 vs 2.3	< 0.05 < 0.05 < 0.05 < 0.05	6 vs 4
Shan, 2006	62 (59.68%)	61	Qianggu capsule, 1 capsule/time, 3 times/day, 12wks	α-D3 capsule, 1 capsule/time, twice/day, 12wks	BMD (LS) BMD (femoral) Total effectiveness rate	1.038 vs 0.936 0.7961 vs 0.7395 90.63 vs 86.67	> 0.05 < 0.01 > 0.05	2 vs 0
**3. Qing’e pill**, main ingredients: cortex eucommiae, fructus psoraleae, walnut, and garlic.								
Niu, 2012	39 (100%)	47–61	Qing’e pill, 9 g/time, 2 times/day, 8wks	Caltrate D, 600 mg, once/day, 8wks	BMD (LS/femoral) Total effectiveness rate	−2.04 vs −2.06 (Z socre) 90 vs 89	< 0.01 < 0.05	NR
**4. Xianling Gubao capsule (XLGB)**								
Jin, 2014	160 (59.38%)	70	XLGB capsule, 0.5 g, 3 capsules/time, twice/day, 6wks	Caltrate D, 600 mg, twice/day, 6wks	BMD (LS) BMD (femoral) Total effectiveness rate	0.8 vs 0.74 0.79 vs 0.77 91.25 vs 77.5	< 0.05 > 0.05 < 0.05	NO
Qin, 2015	160 (55%)	72	XLGB capsule, 0.5 g, 2 capsules/time, 3times/day, 24wks	Calcium D, 1 tablet/time, twice/day, 24wks	BMD (LS) BMD (femoral)	0.786 vs 0.742 0.673 vs 0.651	< 0.05 < 0.05	NR
Wu, 2013	84 (33.33%)	61	XLGB capsule, 0.5 g, 1 capsule/time, twice/day, 6wks	Calcium carbonate vitamin D3 tablet, 500 mg, twice/day, 6wks	BMD (LS) Total effectiveness rate	0.682 vs 0.639 95.24 vs 71.43	< 0.05 < 0.05	0 vs 7
Zheng, 2019	98 (80.61%)	56	XLGB capsule, 0.5 g, 3 capsules/time, twice/day, 24wks	Calcium carbonate vitamin D3 tablet, once/day, 24wks	BMD (LS) Total effectiveness rate	0.82 vs 0.74 83.7 vs 73.8	< 0.05 < 0.05	NO
Le, 2020	80(33.75%)		XLGB capsule, 0.5 g, 3 capsules/time, twice/day, 24wks	Alendronate, 70 mg, once/7 days, 24wks	BMD (LS) BMD (femoral) VAS	0.89 vs 0.70 0.79 vs 0.62 2.01 vs 3.09	< 0.05 < 0.05 < 0.05	NR
Lin, 2017	60 (65.00%)	61	XLGB capsule, 0.5 g, 3 capsules/time, twice/day, 4wks	Calcium carbonate vitamin D3 tablet, 500 mg, twice/day, 4wks	BMD (LS) Total effectiveness rate	0.634 vs 0.867 96.67 vs 73.33	< 0.05 < 0.05	1 vs 4
Xu, 2009	104 (100%)	58	XLGB capsule, 0.5 g, 1 capsule/time, 3times/day, 24wks	Alendronate, 70 mg, once/7 days, 24wks	BMD (LS) BMD (femoral)	0.740 vs 0.740 0.440 vs 0.441	> 0.05 > 0.05	3 vs 3
**5. Liuwei Dihuang pill**								
Zhang, 2011	60 (75.00%)	59	Liuwei Dihuang pill, 8 pills/time, 3times/day, 48wks	Calcium carbonate vitamin D3 tablet, 0.6 g, once/day, 48wks	BMD (LS) BMD (femoral)	0.771 vs 0.733 0.633 vs 0599	< 0.01 < 0.01	NO
Guan, 2006	40 (100%))	57	Liuwei Dihuang pill, 8 pills/time, 3times/day, 24wks	Caltrate D, 0.6 g, once/day, 24wks	BMD (LS) BMD (femoral) BMD (radius) Total effectiveness rate	0.661 vs 0.627 0.580 vs 0.531 0.545 vs 0.512 85 vs 60	< 0.05 < 0.05 < 0.05 < 0.05	NO
Wei, 2012	100 (100%)	56	Liuwei Dihuang pill, 8 pills/time, 3 times/day, 24wks	Caltrate D, 1 tablet/time, 3times/day, 24wks	BMD	0.90 VS 0.88	< 0.05	3 vs 2
Ma, 2011	71 (100%)	55	Liuwei Dihuang pill, 8 pills/time, 3 times/day, 24wks	Caltrate D, 600 mg, 3times/day, 24wks	BMD (LS)	0.94 vs 0.89	< 0.05	2 vs 3
Zhang, 2003	42 (100%)	64	Liuwei Dihuang pill, 8 pills/time, 3 times/day, 48wks	Calcium, 500 mg, 3times/day, 48wks	BMD (radius) BMD (ulna)	0.045 vs 0.004 0.051 vs 0.032	< 0.05 < 0.05	NR
**6. Zuogui pill**								
Li, 2018	200 (NR)	65	Zuogui pill and Yougui pill, twice/day, 24wks	Placebo, twice/day, 24wks	BMD (LS) BMD (femoral) VAS pain	0.79 vs 0.74 0.81 vs 0.78 2.2 vs 3.4	< 0.05 < 0.05 < 0.05	3 vs 2
Sun, 2002	90 (58%)	65	Jawei Zuogui pill, 3 g, 3times/day, 12wks	Calcium granule, 5 g, 3times/day, 12wks	BMD (LS) Total effectiveness rate	0.78 vs 0.68 91.67 vs 50.00	< 0.05 < 0.05	NR
**7. Gusongbao capsule**								
Zou, 2012	80 (61.25%)	49–72	Gusongbao capsule, 3 capsules, twice/day, 4wks	Calcium carbonate D1 tablets, twice/day, 4wks	BMD (LS) BMD (femoral) BMD (radius) Total effectiveness rate	0.76 vs 0.72 0.8 vs 0.7 0.4 vs 0.33 100 vs 84	< 0.05 < 0.05 < 0.05 < 0.05	NR
He, 2010	100 (62%)	62	Gusongbao capsule, 2 capsules/time, 3 times/day, 24wks	Calcium D, 600 mg, once/day, 24wks	BMD (LS) BMD (femoral) BMD (radius)	0.875 vs 0.815 0.716 vs 0.670 0.386 vs 0.341	< 0.05 < 0.05 < 0.05	NR
He, 2016	46 (54.35%)	41	Gusongbao capsule, 2 capsules/time, 3 times/day, 24wks	Calcium D, 2 capsules/time, 3 times/day, 24wks	BMD (LS)	0.884 vs 0.812	< 0.05	NR
Liu, 2020	66(68.18%)	41	Gusongbao capsule, 1 g/time, 3 times/day, 24wks	Calcium Carbonate D3, 1.2 g/time, 3 times/day, 24wks	BMD	0.876 vs 0.812	< 0.01	0 vs 4

NR, not reported; NO, not occurred.

(1) BMD = Bone mineral density, BMD (LS) = BMD (lumbar spine).

(2) BMD (femoral): includes BMD (femoral neck), BMD (wards area), BMD (femoral great trochanter), and BMD (Totsl).

(3) VAS pain: 0–10, lower score = better outcome.

(4) Total effectiveness rate (%) was determined as the quotient of the number of cured and improved patients divided by the total number of patients.

**Table 2.** Summary of evidence and effects of CPM interventions for osteoporosis

Study Characteristic	No. of Studies
**Main varieties**	
Xianling Gubao capsule	7
Qianggu capsule	6
Jintiange capsule	5
Liuwei Dihuang pill	5
Gusongbao capsule	4
Zuogui pill	2
Qing’e pill	1
**Outcomes**	
BMD (lumbar spine)	22 (18+,4-)
BMD (femoral)	15 (12+,3-)
BMD (radius)	6 (5+,1-)
BMD (ulna)	2 (2+)
BMD	4 (3+,1-)
VAS pain score	3 (3+)
Total effectiveness rate	14 (13+,1-)
Adverse events	18 (10+,8-)

+ overall beneficial effect; – no effect

The experimental groups contained 7 CPM, including Jintiange capsule (5 studies), Qianggu capsule (6 studies), Qing’e pill (1 study), Xianling Gubao capsule (7 studies), Liuwei Dihuang pill (5 studies), Zuogui pill (2 studies), and Gusongbao capsule (4 studies). The China Food and Drug Administration (CDFA) classified all medications as proprietary. An overview of CPM components utilized in OP is provided in Table 3. For 4–48 weeks, the CPM was administered orally one to three times per day. The control groups received calcium (21 studies), alpha calcidol (2 studies), alendronate (5 studies), tibolone tablet (1 study), and placebo (1 study) as therapies.

**Table 3.** Overview of ingredients of CPM for osteoporosis

Main varieties	Drug composition (Chinese pinyin/Latin name)	Approval number of SFDA	Prescription functions (TCM patterns)
**Jintiange capsule**	Artificial tiger bone meal.	Z20030080	Strengthen the bones
**Qianggu capsule**	The total flavonoids of Rhizoma Drynariae (Gusuibu, *Davallia mariesii Moore ex Bak.).*	Z20030007	Replenish the kidney, strengthen the bones, and relieve pain
**Qing’e pill**	Cortex Eucommia (Duzhong, *Eucommia ulmoides Oliv*.), Fructus Psoraleae (Buguzhi, *Psoralea corylifolia Linn*.), Walnut Kernel (Hetaoren, *Juglans regia*.), Allium Garlic (Dasuan, *Allium sativum L*.).	Z32020099	Tonify kidney, strengthen the bones
**Xianling Gubao capsule**	Herba epimedii (Yinyanghuo, *Epimedium brevicornu Maxim*.), Radix Dipsaci (Xuduan, *Dipsacus asper Wall.ex Henry*), Fructus Psoraleae (Buguzhi, *Psoralea corylifolia Linn*.), Radix Rehmanniae (Dihuang, *Rehmannia glutinosa Libosch*.), Radix Salviae miltiorrhizae (Danshen, *Salvia miltiorrhiza Bge*.), Rhizoma Anemarrhena (Zhimu, *Anemarrhena asphodeloides Bge*.).	Z20025337	Tonify the liver and kidney, promote blood circulation, remove blood stasis, and strengthen the bones
**Liuwei Dihuang pill**	Rehmannia glutinosa (Dihuang, *Rehmannia glutinosa (Gaetn.) Libosch. ex Fisch. et Mey*.), Fructus corni (Shanzhuyu, *Cornus officinalis Sieb. et Zucc*.), Rhizoma dioscoreae (Shanyao, *Dioscorea oppositifolia L*.), Cortex moutan (Danpi, *Paeonia suffruticosa Andr*.), Tuckahoe (Fuling, *Poria cocos(Schw.) Wolf*), Rhizoma alismatis (Zexie, *Alisma orientalis (Sam.) Juzep*.)	Z19993068	Nourish both yin and kidney
**Zuogui pill**	Rehmannia glutinosa (Dihuang, *Rehmannia glutinosa (Gaetn.) Libosch. ex Fisch. et Mey*.), Semen cuscutae (Tusizi, *Cuscuta chinensis Lam*.), Twotooth achyranthes root (Niuxi, *Radix Achyranthis Bidentatae*), Tortoise-plastron glue (Guibanjiao, *Colla Carapacis et Plastri Tes*), Deerhorn Glue (Lujiaojiao, C*olla Cervi Cornus*), Rhizoma dioscoreae (Shanyao, *Dioscorea oppositifolia L*.), Fructus corni (Shanzhuyu, *Cornus officinalis Sieb. et Zucc*.), Wolfberry fruit (Gouqi, *Fructus Lycii*).	Z41020696 Z11020735	Nourish both yin and kidney
**Gusongbao capsule**	Herba epimedii (Yinyanghuo, *Epimedium brevicornu Maxim*.), Red Peony Root (Chishao, *Radix Paeoniae Rubra*), Rhizoma Sparganii (Sanleng, *Sparganium stoloniferum Buch.-Ham*), Curcuma zedoaria (Ezhu, *Rhizoma Curcumate zedoariae*), Dried rehmannia root (Shengdihuang, *Rehmannia glutinosa Libosch*.), Rhizoma Anemarrhenae (Zhimu, *Anemarrhena asphodeloides Bunge*), Radix Dipsaci (Xuduan, *Dipsacus asper Wall.ex Henry*), Szechuan Lovage Rhizome (Chuanxiong, *Ligusticum chuanxiong Hort*.), Oyster Shell (Muli, *Concha Ostreae*).	Z20030084	Tonify the kidney, promote the blood circulation, strengthen the bones and gluten

### Quality assessment

**Figure 2.**
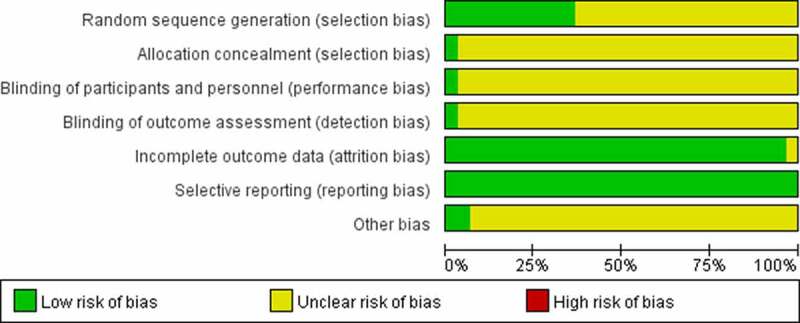
Risk of bias distribution graph.

**Figure 3.**
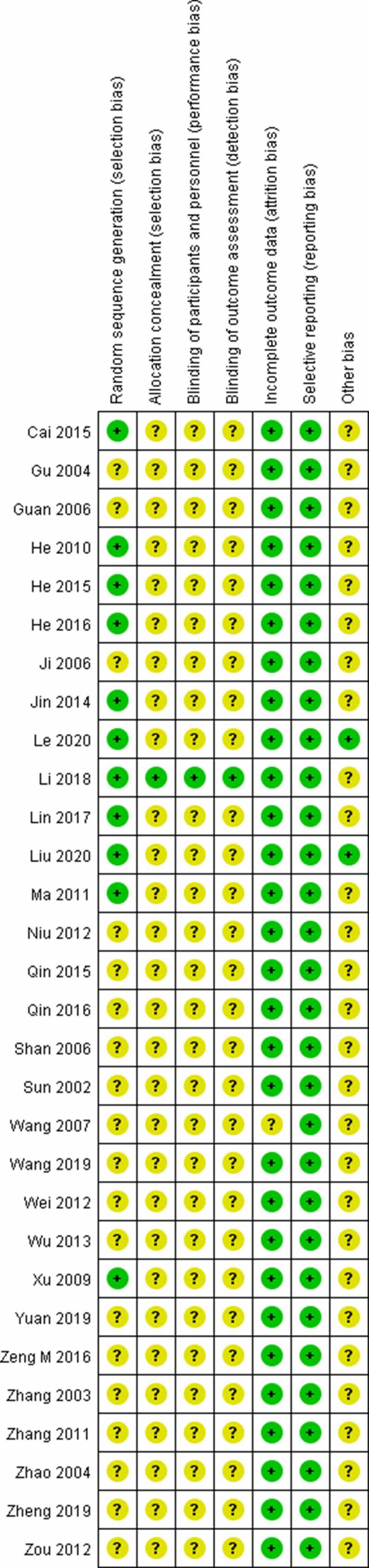
Risk of bias summary.

The quality (risk of bias) assessment of trials was performed using a modified version of The Cochrane Collaboration’s tool [51]. Figure 2 and Figure 3 depict the risk of bias distribution and research quality within this evidence base. Overall, the trials’ bias quality was modest. In 11 studies (36.67%), randomization was satisfactory, but in 19 trials (63.33%), it was questionable. Although one research reported satisfactory allocation concealment, the remaining 29 trials (96.67%) were ambiguous. Blinding of participants and personnel happened in 1 trial (3.33%), but was unclear in 2 trials (6.67%) and high risk in 27 trials (90%). Blinding of the outcome happened in 1 trial (3.33%) but was unclear in the other 29 (96.67%). All studies reported the similarity of study groups at the baseline (100%). There was no study that mentioned selective reporting.

### Meta-analysis

We used the BMD to assess the quantitative treatment effects in the 30 eligible RCTs.

**Figure 4.**
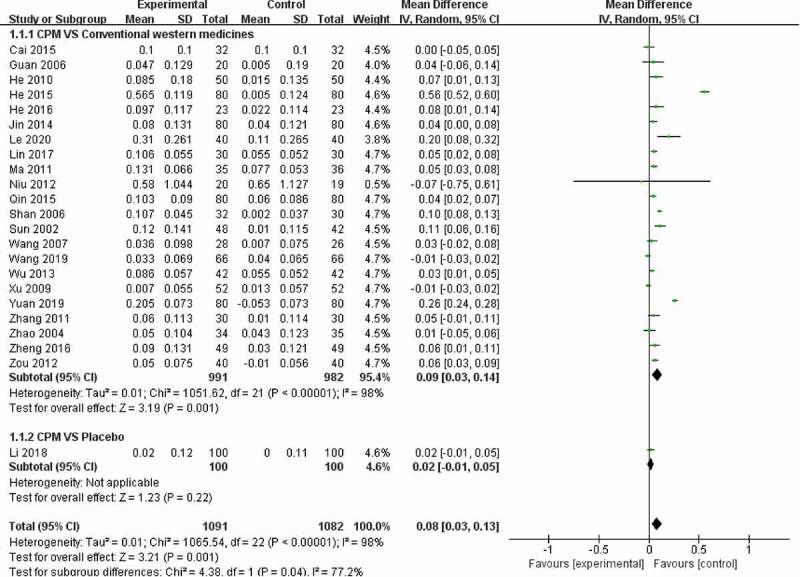
Effect of CPM therapy on BMD (lumbar spine).

**Figure 5.**
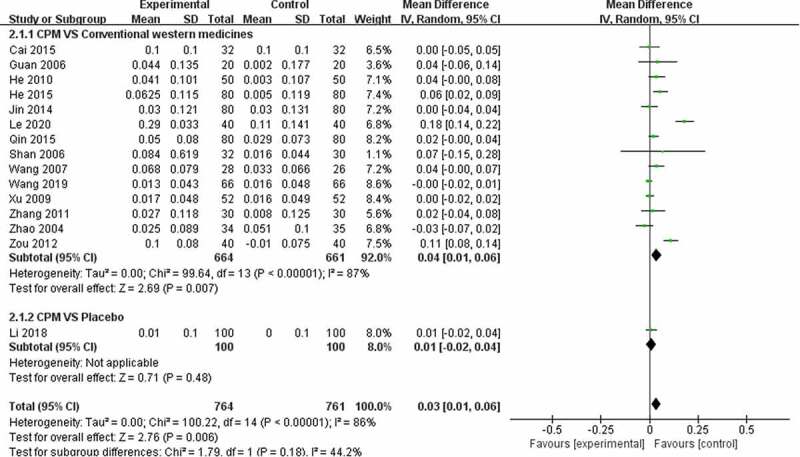
Effect of CPM therapy on BMD (femoral).

**Figure 6.**
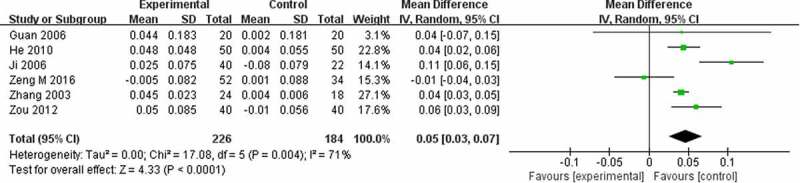
Effect of CPM therapy on BMD (radius).

**Figure 7.**
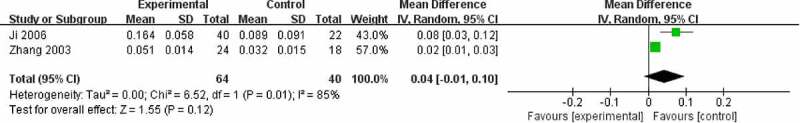
Effect of CPM therapy on BMD (ulna).

**Figure 8.**
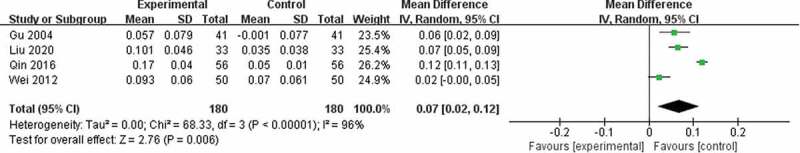
Effect of CPM therapy on BMD.

**Figure 9.**
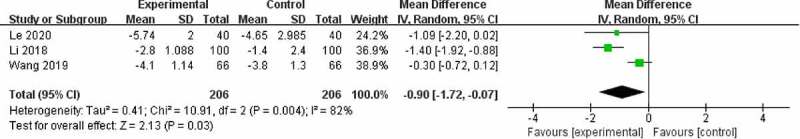
Effect of CPM therapy on VAS pain score.

**Figure 10.**
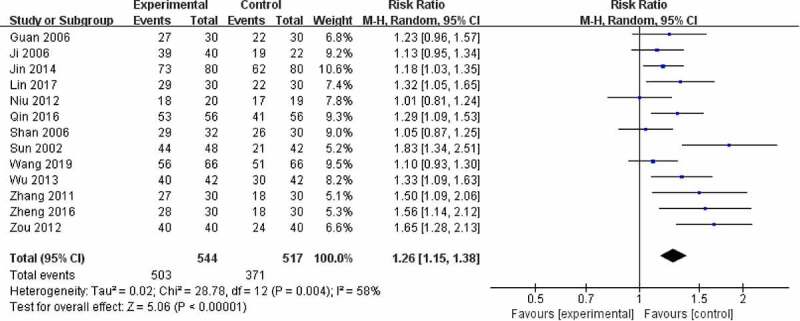
Effect of CPM therapy on total effectiveness rate.

Twenty-three trials used BMD (lumbar spine) (Figure 4), 15 trials used BMD (femoral) (FIGURE 5), 6 trials used BMD (radius) (FIGURE 6), 2 trials used BMD (ulna) (Figure 7), and **4** trials used BMD (Figure 8). At the same time, 5 trials used the VAS pain score (Figure 9) to measure the pain levels, and 14 trials assessed overall pain, physical performance, and wellness using the total effectiveness rate (Figure 10).

#### BMD (lumbar spine)

Twenty-three trials involving 2173 patients were used to perform a meta-analysis of clinical efficiency using BMD (lumbar spine). The heterogeneity (*I*^2^) score of BMD (lumbar spine) was high. The results of the random-effects meta-analysis indicated that patients in the CPM groups had significantly higher BMD (lumbar spine) than those in the control groups of calcium, alpha calcidol, and alendronate (MD = 0.08; 95% CI, 0.03–0.13) after 4 to 48 weeks of treatment. Further subgroup analysis exploring the improvement of different controls on BMD (lumbar spine) showed that CPM therapy had a better effect compared with conventional western medicines (MD = 0.09; 95% CI, 0.03–0.14), and there was no difference between CPM and placebo control groups (MD = 0.02; 95% CI, −0.01–0.05) after 24 to 48 weeks of treatment (Figure 4).

#### BMD (femoral)

Fifteen trials involving 1525 patients were used to perform a meta-analysis of clinical efficiency using BMD (femoral). The results of the random-effects meta-analysis indicated that patients in the CPM groups had significantly higher BMD (femoral) than those in the control groups of calcium, alpha calcidol, and alendronate (MD = 0.03; 95% CI, 0.01–0.06) after 4 to 36 weeks of treatment. The heterogeneity (*I*^2^) score of BMD (femoral) was 86%. Further subgroup analysis exploring the improvement of different controls on BMD (femoral) showed that CPM therapy has a better effect compared with conventional western medicines (MD = 0.04; 95% CI, 0.01–0.06), and there was no difference between CPM group and placebo control groups (MD = 0.01; 95% CI, −0.02–0.04) after 12 to 48 weeks of treatment (Figure 5).

#### BMD (radius)

Six trials evaluated clinical efficiency using BMD (radius), involving 410 patients and 4 CPMs. The results of the random-effects meta-analysis indicated that the BMD (radius) elevation in the CPM group was much more significant than the group taking conventional western medicines (MD = 0.05; 95% CI, 0.03–0.07) after 4 to 48 weeks of treatment. (Figure 6).

#### BMD (ulna)

Two trials additionally evaluated clinical efficiency using BMD (ulna), involving 104 patients and 2 CPMs. The results of the random-effects meta-analysis indicated that there was no difference between the CPM group and calcium (MD = 0.04; 95% CI, −0.01–0.10) after 12 to 48 weeks of treatment, what suggested the improvement effect on BMD was very weak (Figure 7).

#### BMD

Four trials involving 360 patients and were used to perform a meta-analysis of clinical efficiency by BMD of unspecified site. With a very high heterogeneity (*I^2^*) score, the results of the random-effects meta-analysis indicated that patients in the CPM groups had significantly higher BMD than conventional western medicines (SMD = 1.67; 95% CI, 0.26–3.08) after 12 to 24 weeks of treatment (Figure 8).

#### VAS pain score

To investigate the improvement effect of CPM on pain in patients, we extracted VAS scores from three trials including 412 patients. The results of the random-effects meta-analysis indicated that patients in the CPM groups had significantly lower pain scores than the groups taking conventional western medicines (MD = −0.90; 95% CI, −1.72 – −0.07) after 24 to 48 weeks treatment, which means CPM does relieve pain in OP patients. (Figure 9).

### Total effectiveness rate

**Figure 11.**
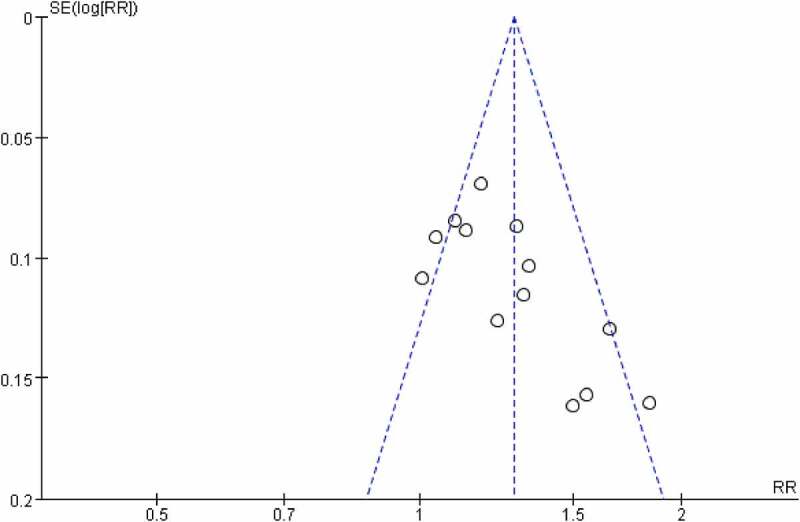
Funnel plots for publication bias.

Thirteen trials involving 1061 patients assessed the overall response of CPM in patients using the total effectiveness rate compared to conventional western medicine. The overall clinical effectiveness rate in the CPM groups was 92.46% (risk ratio [RR] = 1.26; 95% CI, 1.15 to 1.38), with a high degree of heterogeneity (*I*^2^ = 58%). Our meta-analysis outcome showed that CPM therapy of 4 to 48 weeks could improve clinical symptoms including overall pain, physical performance, and wellness for patients with OP (Figure 10). Funnel plot suggests that there might be publication bias (Figure 11).

### Adverse events

Eighteen trials provided information on adverse events, while 12 trials did not. Of the 18 trials, 10 reported that 34 patients had adverse events in the CPM group and 48 patients had adverse events in the control group, and 7 trials reported that no adverse events occurred. The reported minor adverse events included dry mouth, constipation, abdominal distension, diarrhea, gastrointestinal discomfort, rash, nausea, vomiting, and muscle soreness. No serious adverse events occurred in the CPM group, but Zhao et al. stated that three patients experienced vaginal bleeding in the control group [23]. The incidence of adverse events in the CPM group was less than that in the control group, and the adverse events disappeared after stopping medication.

## Discussion

The results of our meta-analysis indicate that CPM therapy is more effective than general oral medicine or placebos in relieving symptoms and improving the BMD of OP and does not pose significant safety risks. Overall, CPM therapy appears to be safe and effective for people who suffer from OP.

The functional imbalance of osteoblasts and osteoclasts can directly lead to bone loss. In women, postmenopausal decline in estrogen levels is critical to the pathogenesis of OP, in addition to calcium and vitamin D deficiencies can also accelerate this process [52,53]. The management of OP focuses on two tasks: prevention and treatment – both of which Chinese medicine can play a role. On the one hand, the specific components contained in Chinese herb medicine play a key role in bone metabolism. On the other hand, on the basis of Chinese Medicine theory, the required Chinese herb medicines mainly tonify kidney (Shen) and spleen (Pi), strengthen bones, improve cell metabolism, and invigorate Qi and blood. The Guidelines for the Diagnosis and Management of Primary Osteoporosis (2017) in China recognize the total flavonoids of Drynariae, icariin, and artificial tiger bone meal as the ingredients of Chinese medicine with anti-osteoporosis pharmacological effects. The Guidelines also list the Qing’e pill, Liuwei Dihuang pill, Zuogui pill, and Yougui pill as recommended drugs^54^.

A growing body of evidence is beginning to shed light on the potential biological mechanisms through which CPM therapy works in OP. Various clinical trials and animal studies of different kinds of CPM have demonstrated that kidney-tonifying Chinese herbal medicine can prevent and treat bone loss by increasing bone density, promote bone resorption decreased the level of urine Ca/Cr [55]. The primary ingredient in the Jintiange capsule, artificial tiger bone meal, contains a variety of trace elements and amino acids essential for bone production. The total flavonoids of Rhizoma Drynariae in the Qianggu capsule, and the icariin, fructus psoraleae, radix dipsaci, and rehmannia glutinosa in other CPMs all directly boost blood calcium levels and stimulate bone cells [56,57]. According to the findings of this study, CPMs can dramatically improve BMD and are more effective than alendronate, calcium, and vitamin D. In addition, the radix salviae miltiorrhizae in Xianling Gubao capsule contains tanshinone, as does the twotooth achyranthes root, which is the principal element in the Zuogui Pill and contains complete achyranthes saponins. Both of these lessen the VAS score by acting anti-inflammatory, analgesic, and blood flow improvers.

Furthermore, numerous studies indicate that CPM may have the anti-osteoporosis benefits in OP patients via a variety of targets and pathways. According to certain research, the Zuogui pill can prevent OP by rectifying the imbalance of bone formation and bone resorption through different targets and pathways, including Wnt1, LRP-5, Wnt β-catenin, and TGF-β-Smad signal [58,59,]. The network pharmacology analysis approach demonstrated that Xianling Gubao had a therapeutic impact by the regulation of osteoclastic differentiation modulation, inhibition of inflammatory responses, and involvement hub genes (AKT1, MAPK1, MAPK8, TP53, and STAT3) [60]. Some studies of the Liuwei Dihuang pill investigations hypothesized that some genes may play critical roles in OP therapeutic processes, including ATF2, FBXW7, RDX, NCOA3, TCF4, DUSP6, PELI2, and STX7 [61,62,]. It may also have effects through the up-regulation of cardiotrophin-like cytokine factor 1 (CLCF1) gene expression and activating the Janus kinase/signal transducer and activator of transcription (JAK/STAT) signaling pathway [63]. In addition, a previous study that focused on holistic quality control in a specific kind of CPM provided valuable information for guaranteeing the safety, effectiveness, and controllability of CPM therapy [64]. Overall, further research is warranted to explore the underlying biological mechanisms of CPM therapy for OP.

The most serious risk about using Chinese medicine is its toxicity and adverse effects. Several studies have found that the adverse responses experienced by OP patients receiving CPM are quite mild and can be alleviated by stopping CPM or using symptomatic therapy. As a result, whether compared to oral calcium, vitamin D, or Alendronate, CPMs have no extra adverse effects and pose minimal harm to patients. This might be an advantage of CPM.

These findings are consistent with a number of recent reviews of CPM therapy. Jing Sun et al., for example, revealed that CPM was a favorable choice for treating patients with OP in terms of increasing BMD, decreasing pain, and lowering adverse events in 31 trials utilizing Jintiange capsules alone and in conjunction with other medications[65]. Another review of 10 trials by Xu Wei et al. suggested that Qianggu capsules were associated with the improvement of BMD for primary OP[66]. In addition, 3 reviews have shown that Xianling Gubao capsules are effective in improving BMD and serum calcium levels, increasing the clinical effectiveness rate, and reducing pain [67–69]. Indeed, current research on CPM therapy and its capacity to enhance BMD, reduce pain, and raise clinical effectiveness rates in OP supports our findings.

Our study has limitations, despite its merits. For starters, several of the included RCTs have a significant risk of bias. There has only been one double-blinding and placebo-controlled trial recorded. Second, treatment durations vary amongst studies, spanning from 1 to 12 months; consequently, lengthier and more consistent follow-ups will be warranted in future research. Third, there are many kinds of TCMP utilized in clinical practice to treat OP, but only 11 types were included in this study. We also observed high heterogeneity due to diverse kinds, formulations, and control groups. Lastly, despite CPM’s statistically significant effects on BMD and symptom improvement in OP patients, the clinically essential advantages of CPM therapy remain to be determined. Thus, the potential benefits of CPM for OP need to be further evaluated through high-quality clinical trials with more rigorous methodologies.

## Conclusions

The results of this meta-analysis indicate that CPM therapy may be a valuable treatment regimen for OP by improving BMD and symptoms while reducing the risk of adverse events, but it is a pity that the quality of trials included is moderate. Due to this deficiency, more rigorously designed and well-controlled RCTs are warranted to support the clinical application of CPM therapy for OP patients. Future clinical research should focus on their potential to reduce these patients’ risks of serious adverse events.

## Abbreviations

TCM: traditional Chinese medicine
CPM: Chinese patent medicine
OP: osteoporosis
RCTs: randomized controlled trials
BMD: bone mineral density
MD: mean difference
CI: confidence interval
RR: risk ratio
VAS: visual analogue scale
CDFA: China Food and Drug Administration.
